# The Effects of Selection

**Published:** 1915-12

**Authors:** 


### The Effects of Selection.

Recent experiments at the Illinois Agricultural Experiment Station are likely to prove of great value in showing the effect of selection as applied to the development of new cereals, to say nothing of its added value to agriculture. These experiments, lasting over a period of seventeen years, in some instances, show how, from a given variety of corn, a strain has been developed increasing the protein content from 10.9 per cent to 14.83 per cent, and by selecting for a low protein content a strain has been developed containing as low a percentage as 7.71 per cent.

From the same variety, selecting for the oil content, resulted in producing a strain four times as rich in this property.

Selecting for the height an ear would form on the stalk, a difference of four and one-half feet was obtained. Likewise, selection was made for the angle at which an ear would grow on the stalk with the development of a definite variety showing this quality. Next, the attempt was made to grow two ears to the stalk instead of one, which has also succeeded favorably.

Not only have these physical characteristics been modified by selection, but a deeper insight into the nature of the transmission of unite characters have been investigated, at the same station, which has resulted in further proving the universality of the Mendelian principles of inheritance. This is done by hybridizing varieties of diverse characters, resulting in separate independently transmissible characters.

Other cereal products are being experimented with, especially the soy bean, which by selection will be made to develop a variety rich in oil production and another suitable for human food.

